# Differential Distribution of Retinal Ca^2+^/Calmodulin-Dependent Kinase II (CaMKII) Isoforms Indicates CaMKII-β and -δ as Specific Elements of Electrical Synapses Made of Connexin36 (Cx36)

**DOI:** 10.3389/fnmol.2017.00425

**Published:** 2017-12-19

**Authors:** Stephan Tetenborg, Shubhash C. Yadav, Sheriar G. Hormuzdi, Hannah Monyer, Ulrike Janssen-Bienhold, Karin Dedek

**Affiliations:** ^1^Animal Navigation/Neurosensorics, Institute for Biology and Environmental Sciences, University of Oldenburg, Oldenburg, Germany; ^2^Division of Neuroscience, Ninewells Hospital and Medical School, University of Dundee, Dundee, United Kingdom; ^3^Cancer Research Center (DKFZ), Heidelberg, Germany; ^4^Visual Neuroscience, Department of Neuroscience, University of Oldenburg, Oldenburg, Germany; ^5^Research Center Neurosensory Science, University of Oldenburg, Oldenburg, Germany

**Keywords:** CaMKII, gap junction, electrical synapse, connexin36, amacrine cell, bipolar cell, retina

## Abstract

AII amacrine cells are essential interneurons of the primary rod pathway and transmit rod-driven signals to ON cone bipolar cells to enable scotopic vision. Gap junctions made of connexin36 (Cx36) mediate electrical coupling among AII cells and between AII cells and ON cone bipolar cells. These gap junctions underlie a remarkable degree of plasticity and are modulated by different signaling cascades. In particular, Ca^2+^/calmodulin-dependent protein kinase II (CaMKII) has been characterized as an important regulator of Cx36, capable of potentiating electrical coupling in AII cells. However, it is unclear which CaMKII isoform mediates this effect. To obtain a more detailed understanding of the isoform composition of CaMKII at retinal gap junctions, we analyzed the retinal distribution of all four CaMKII isoforms using confocal microscopy. These experiments revealed a differential distribution of CaMKII isoforms: CaMKII-α was strongly expressed in starburst amacrine cells, which are known to lack electrical coupling. CaMKII-β was abundant in OFF bipolar cells, which form electrical synapses in the outer and the inner retina. CaMKII-γ was diffusely distributed across the entire retina and could not be assigned to a specific cell type. CaMKII-δ labeling was evident in bipolar and AII amacrine cells, which contain the majority of Cx36-immunoreactive puncta in the inner retina. We double-labeled retinas for Cx36 and the four CaMKII isoforms and revealed that the composition of the CaMKII enzyme differs between gap junctions in the outer and the inner retina: in the outer retina, only CaMKII-β colocalized with Cx36-containing gap junctions, whereas in the inner retina, CaMKII-β and -δ colocalized with Cx36. This finding suggests that gap junctions in the inner and the outer retina may be regulated differently although they both contain the same connexin. Taken together, our study identifies CaMKII-β and -δ as Cx36-specific regulators in the mouse retina with CaMKII-δ regulating the primary rod pathway.

## Introduction

Electrical synaptic transmission allows fast propagation of electrical impulses, cell synchronization and network oscillations (Hormuzdi et al., 2001; Christie et al., 2005). It relies on specialized cell-cell contacts called gap junctions, which are formed by connexin proteins. Compared to chemical synapses, electrical synapses have long been considered rather static. However, recent evidence indicates that the efficacy of these synapses is dynamically regulated by neuromodulators and interaction with glutamatergic synapses (reviewed in Pereda et al., 2013). Both regulatory pathways activate signaling cascades that change the phosphorylation state of the underlying connexin protein.

Connexin36 (Cx36) is the predominant isoform in the central nervous system and is strongly expressed in the retina, where it forms electrical synapses essential for visual signal transmission (Bloomfield and Völgyi, 2009). The vast majority of retinal Cx36 is expressed in AII amacrine cells. These interneurons act as relays and utilize gap junctions to transmit rod-evoked signals into the cone pathway (Mills and Massey, 1995; Veruki and Hartveit, 2002). In the absence of Cx36, this pathway is abolished and Cx36-deficient mice completely lose rod-mediated ganglion cell responses (Deans et al., 2002) and show severe defects in scotopic vision (Güldenagel et al., 2001; Deans et al., 2002). The electrical synapses formed by the AII amacrine cells are highly dynamic and coupling changes with different levels of background illumination (Bloomfield and Völgyi, 2004), with low coupling in darkness and under photopic conditions and strong coupling at higher scotopic light levels. Photopic conditions lead to an increase in dopamine levels and activate a signaling cascade in AII cells by which protein phosphatase 2A (PP2A) dephosphorylates serine 293 of Cx36, leading to a decrease in gap junction coupling (Kothmann et al., 2009). At higher scotopic light levels, in contrast, the same amino acid is phosphorylated, leading to an increase in coupling. The phosphorylation is mediated by Ca^2+^/calmodulin-dependent kinase II (CaMKII), which is activated by Ca^2+^ entering the AII cell through extrasynaptic NMDA receptors (Kothmann et al., 2012). Thus, in AII cells, CaMKII potentiates Cx36 coupling in an activity-dependent manner.

CaMKII is a well-described multifunctional enzyme complex, capable of translating neuronal activity into changes in synaptic efficiency. Its molecular structure is based on two hexagonal rings which contain 12 subunits, each gaining catalytic activity after binding of Ca^2+^/calmodulin (Lisman et al., 2002). The impact of CaMKII on electrical transmission just emerged in the last 10 years, yet it became apparent that its interaction with Cx36 is conserved across species (Alev et al., 2008; Flores et al., 2010). However, most observations focused on the physiological effects of the enzyme rather than the contribution of its four isoforms: CaMKII-α, -β, -γ and -δ (Del Corsso et al., 2012; Kothmann et al., 2012). These variants derive from four different genes and share a common protein structure with an N-terminal catalytic domain, a calmodulin-binding domain, and a C-terminal association domain, which enables oligomerization of all subunits (Lisman et al., 2002). Despite their strong degree of homology, CaMKII isoforms are differentially expressed and serve distinct functions: CaMKII-α and -β represent neuron-specific isoforms; CaMKII-β possesses an actin-binding domain, providing essential functions for synapse formation and dendritic morphology (Okamoto et al., 2007; Kim et al., 2015). CaMKII-γ and -δ, however, are ubiquitously expressed and have been characterized as important regulators of Ca^2+^-homeostasis in the heart (Weinreuter et al., 2014).

In order to obtain a more detailed view on CaMKII signaling at Cx36-containing gap junctions in the vertebrate retina, we analyzed the distribution of all four CaMKII isoforms. We found that only CaMKII-β and -δ colocalized with Cx36. This colocalization was cell type-specific: CaMKII-δ was expressed at AII-amacrine cell gap junctions, whereas CaMKII-β associated with Cx36 at the dendritic tips of OFF cone bipolar cells. Thus, our results indicate that the CaMKII subunit composition differs between electrical synapses in the outer and inner retina, pointing to different local protein networks that regulate the efficacy of Cx36-containing gap junctions in the retina.

## Materials and Methods

### Animals and Tissue Preparation

All procedures were approved by the local animal care committee (*Niedersaechsisches Landesamt fuer Verbraucherschutz und Lebensmittelsicherheit*) and were in compliance with the guidelines for the welfare of experimental animals issued by the European Communities Council Directive of 24 November 1986 (86/609/EEC) and the laws of the Federal Government of Germany (*Tierschutzgesetz*; BGBl. I S. 1206, 1313 and BGBl. I S. 1934). The experiments in this study were conducted with C57BL/6J, TH::GFP (Matsushita et al., 2002; Knop et al., 2011) and Cx36-EGFP mice (Meyer et al., 2014) at the age of 1–6 month. Mice were deeply anesthetized with CO_2_ and killed by cervical dislocation. Eyes were enucleated and dissected in Ames medium, supplemented with 22 mM NaHCO_3_ and bubbled with carbogen (95%/5% O_2_/CO_2_). The cornea was cut along the ora serrata; thereafter lens and vitreous body were removed. The eyecups were fixed in 2% paraformaldehyde (PFA) in 0.1 M phosphate buffer (PB) for 2 × 10 min. To analyze CaMKII expression in the outer retina, eyecups were dissected and incubated for 30–45 min in Ames medium at 37°C in darkness.

For AII amacrine cell injections in retinal wholemounts, eyes were enucleated, and cornea, lens and vitreous body were removed in oxygenated Ringer’s solution (in mM: 110 NaCl, 2.5 KCl, 1 CaCl_2_, 1.6 MgCl_2_, 10 glucose, 22 NaHCO_3_, adjusted to pH 7.4 with carbogen) at room temperature, as described previously (Meyer et al., 2014). Retinas were isolated from the eyecups and bisected. Each piece was then mounted, ganglion cell side up, on black filter paper (MF, Millipore), which was immersed in 0.0001% DAPI solution for 30–45 min prior to intracellular dye injection.

### Immunhistochemistry

After cryoprotection (immersion in 30% sucrose in PB overnight), the tissue was embedded in TissueTek and cut into 20 μm thick sections. Sections were blocked with 10% normal goat (NGS) or normal donkey serum (NDS) in TBS-Tx (TRIS-buffered saline with 0.3% TritonX-100, pH 7.6). Primary antibodies were diluted in blocking solution and applied at 4°C overnight (Table 1). Secondary antibodies were conjugated to Alexa 488, Alexa 568 or Alexa 647 (1:500, Invitrogen) and applied for 2 h at room temperature. All washing steps were performed with TBS-Tx. Slices were mounted with Vectashield.

**Table 1:** List of primary antibodies used in this study.

Antibody	Host, type	Dilution	Source Cat. (No.)
Bassoon	Mouse, monoclonal	1:500	Enzo Life Sciences, SAP7F405
Cacna1s	Mouse, monoclonal	1:500	Millipore, MAB427
CaMKII-α	Mouse, monoclonal	1:500/1:250 (WM)	Invitrogen, MA1-048
CaMKII-β	Rabbit, polyclonal	1:1000/1:500 (WM)	Abcam, AB34703
CaMKII-γ	Rabbit, polyclonal	1:1000	Abcam, PA5-22168
CaMKII-δ	Rabbit, polyclonal	1:1000/1:500 (WM)	Invitrogen, AB37999
ChAT	Goat, polyclonal	1:100	Chemicon, AB144P
Cx36	Mouse, monoclonal	1:500/1:250 (WM)	Invitrogen, 37-4600
Cx36	Rabbit, polyclonal	1:500	Invitrogen, 51-6300
Cx36	Rabbit, polyclonal	1:500	Invitrogen, 364600
EGFP	Goat, polyclonal	1:500	Rockland, 600-101-215
G0α	Mouse, monoclonal	1:500	Chemicon, MAB3073
PKC-α	Mouse, monoclonal	1:1000	Sigma, Sc-80
PKARII-β	Mouse, monoclonal	1:500	BD Biosciences, 554002
PSD-95	Mouse, monoclonal	1:10000	NeuroMab, 75-028
VGluT1	Guinea pig, polyclonal	1:1000	Millipore, AB5905

*WM, concentration used for retinal whole-mounts*.

After intracellular dye injection, retinal whole-mounts were fixed in 4% PFA in PB for 10 min. Retinas were then incubated with primary antibodies (in PB containing 10% NDS, 0.3% Triton X-100, 0.05% NaN_3_) for 2 days at room temperature. After extensive washing, the retinas were incubated with Alexa Fluor 647-conjugated donkey-anti-rabbit and Alexa Fluor 488-conjugated donkey-anti-mouse secondary antibodies for 1 day at room temperature.

### Intracellular Dye Injections

Dye injections were performed as described previously (Meyer et al., 2014). Briefly, borosilicate glass electrodes were pulled with a Sutter P-97 puller (Sutter, Novato, CA, USA). Electrode tips were filled with 5 mM Alexa Fluor 594 diluted in 0.2 M KCl. Electrodes were then backfilled with 0.2 M KCl. Electrodes typically had resistances between 100 and 200 MΩ. AII amacrine cells were targeted for injection under epifluorescence in the DAPI-stained retina. The dye was iontophoresed with −0.5 nA square pulses of 500 ms at 1 Hz for 5–10 min. The dye was allowed to diffuse for at least 30 min prior to fixation.

### Image Acquisition and Analysis

Images were acquired with a confocal laser scanning microscope (Leica TCS SP8). Stacks of retinal cryosections and whole-mounts were scanned with HC PL APO CS2 63×/1.4 and HC PL APO CS2 40×/1.3 oil objectives, respectively. Pixel size was adjusted for each data set and kept constant for one set of experiments. Stacks were deconvolved, using theoretical point spread functions in the Huygens Essential deconvolution software and processed in Fiji (Schindelin et al., 2012)^1^.

Colocalization of Cx36 and different CaMKII isoforms was analyzed with the *colocalization highlighter* plugin in Fiji and global thresholds. The resulting 8-bit images displaying colocalized puncta of both channels were maximum-projected (6 slices, z-distance 200 nm) and number and area of puncta were measured using the *analyze particle* function in Fiji. We excluded particles with a size of ≤4 square pixels from analysis. The degree of colocalization was expressed as the relative amount of overlapping puncta to total Cx36, measured with the same thresholds. As controls, we analyzed the colocalization in images with one horizontally flipped channel. Per condition, 7–10 regions of interest from at least three different animals were analyzed. All data sets showed normal distribution (tested with a Pearson omnibus normality test in GraphPad Prism 5) and were tested for significance with an unpaired, two-tailed *t*-test. The relative amount of colocalization for all conditions was plotted as bar graph. Similarly, we compared the amount of Cx36, which colocalizes with either CaMKII-β or CaMKII-δ on AII and tyrosine hydroxylase type 2 (TH2) amacrine cells. Here, we first determined the number of Cx36 puncta colocalizing with the dendrites and then determined the number of puncta colocalizing with the respective CaMKII isoform. These analyses were performed in retinal whole-mounts.

Unless stated otherwise, images are presented as single confocal scans adjusted for brightness and contrast for presentation purposes.

## Results

### Differential Distribution of Retinal CaMKII Isoforms

**Figure 1:**
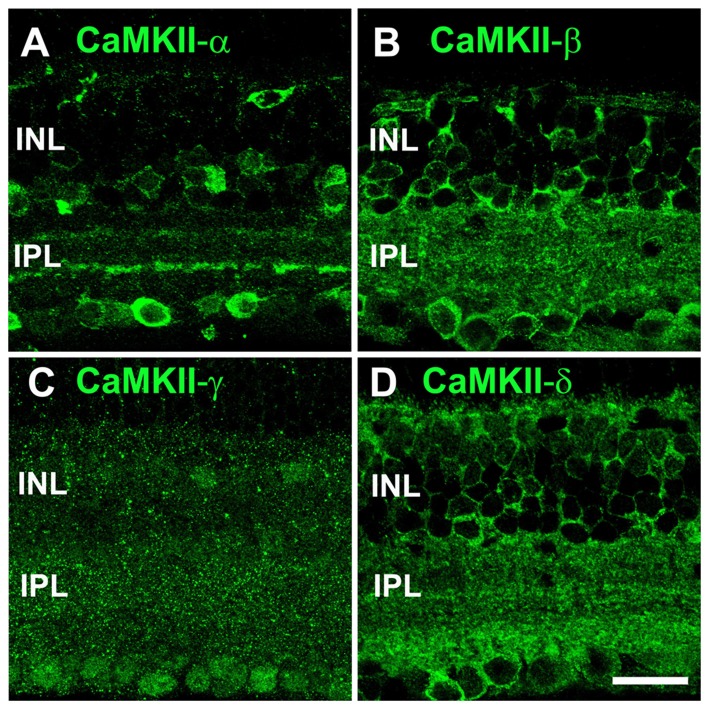
Distribution of all four Ca^2+^/calmodulin-dependent protein kinase II (CaMKII) isoforms in the retina. **(A)** CaMKII-α staining showed outlines of several somata (inner nuclear layer, INL and ganglion cell layer, GCL) and two bands in the IPL.** (B)** CaMKII-β immunoreactivity revealed a punctate pattern in both plexiform layers. CaMKII-β also labeled outlines of neurons in the INL and GCL. **(C)** CaMKII-γ was diffusely distributed across the entire retina. **(D)** CaMKII-δ showed a similar staining pattern as CaMKII-β but its labeling was more intense in the outer plexiform layer (OPL) and proximal IPL. All images are shown as maximum projections of confocal scans (20 optical sections, 0.2 μm thick). Scale: 20 μm.

To study the subunit composition of CaMKII at electrical synapses, we first examined the overall distribution of its four isoforms in the retina (Figure 1). Confocal images revealed a quite distinct distribution for each isoform. Antibodies for CaMKII-α labeled somata in the inner nuclear layer (INL) and ganglion cell layer (GCL), and two prominent bands in the inner plexiform layer (IPL), resembling the ramification pattern of starburst amacrine cells (Figure 1A). A similar distribution has already been described for CaMKII-α in the rat (Ochiishi et al., 1994) and mouse retina (Liu et al., 2000).

Antibodies for CaMKII-β and -δ labeled many puncta in both synaptic layers; however, CaMKII-δ immunoreactivity was more intense than that for CaMKII-β in the outer plexiform layer (OPL) and proximal IPL. Both antibodies also stained the outlines of cells in the INL and GCL (Figures 1B,D). CaMKII-γ immunoreactivity was diffusely distributed across the entire retina and visible in nuclei of neurons in the GCL and INL (Figure 1C). In summary, we reveal a unique distribution pattern for each CaMKII isoform, suggesting that different isoforms may fulfill different functions within the retina. As CaMKII-γ expression appeared ubiquitous, we excluded this isoform as a specific regulator of gap junctions and focused on the remaining three variants in the course of this study.

### CaMKII-α Is Expressed in Starburst Amacrine Cells

**Figure 2:**
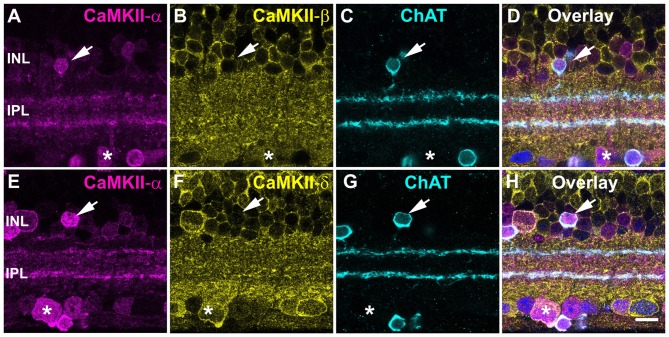
CaMKII-α in starburst amacrine cells. **(A–D)** CaMKII-α but not -β is expressed in starburst amacrine cells (arrow). A ChAT-negative soma coexpressed CaMKII-α and -β (asterisk). **(E–H)** CaMKII-δ was absent in starburst amacrine cells (arrow) but was coexpressed with CaMKII-α in putative ganglion cells (asterisk). To better visualize cell bodies, the overlay shows DAPI labeling in blue. Scale: 10 μm.

The staining pattern for CaMKII-α resembled the stratification and soma distribution of starburst amacrine cells. These cells play a major role in direction selectivity and represent the almost only retinal neurons immunoreactive for choline acetyltransferase (ChAT). Double labeling for CaMKII-α and ChAT indeed showed strong colocalization (Figures 2A,C–E,G,H), consistent with a previous report from rat retina (Ochiishi et al., 1994). CaMKII-α immunoreactivity was most prominent in starburst cells, with stronger labeling in ON than OFF starburst dendrites. However, in the INL and GCL, CaMKII-α-positive but ChAT-negative cells were detected. We also tested for CaMKII-β and -δ expression in starburst amacrine cells (Figures 2B,F) but these two isoforms were absent and instead colocalized with CaMKII-α in some large somata in the GCL (asterisk, Figures 2A–H).

Taken together, these data show that CaMKII-α is the dominant isoform in mouse starburst cells. We did not consider this isoform as a major regulator of gap junctions because: (1) starburst cells are not electrically coupled; (2) CaMKII-α immunoreactivity was only found in a few other cell types; and (3) labeling was not punctate (gap-junction-like).

### Differential Distribution of CaMKII-β and -δ at Glutamatergic Synapses in the IPL

**Figure 3:**
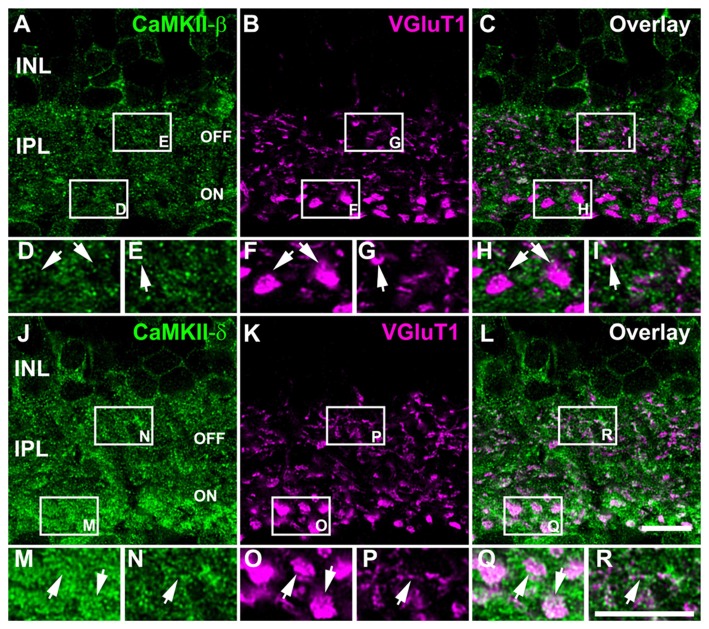
Differential localization of CaMKII-β and -δ at bipolar cell terminals. **(A–C)** Double staining of CaMKII-β and VGluT1. **(D–I)** Magnified images of areas in white boxes **(A–C)** reveal that CaMKII-β immunoreactivity closely surrounded bipolar cell terminals (arrows). **(J–L)** Double staining of CaMKII-δ and VGluT1. **(M–R)** Magnified images indicate strong CaMKII-δ immunoreactivity within proximal (**M,O,Q**, arrows) and distal bipolar cell terminals (**N,P,R**, arrows). Scale: 10 μm.

To analyze whether CaMKII-β and -δ are localized at retinal gap junctions, we studied the expression of both isoforms at bipolar cell terminals because these cell types express different gap junction proteins (Han and Massey, 2005; Maxeiner et al., 2005; Dedek et al., 2006; Hilgen et al., 2011). Double staining for CaMKII and the vesicular glutamate transporter 1 (VGluT1) showed that CaMKII-β immunoreactivity is nearly absent from bipolar cell terminals of the OFF and ON layer. Instead, the labeling closely surrounded bipolar cell terminals, suggesting postsynaptic expression in ganglion and amacrine cells (Figures 3A–I, arrows). This arrangement most likely reflects CaMKIIs function as an effector of Ca^2+^ signals at glutamatergic postsynapses, where the enzyme is concentrated in electron dense structures close to the plasma membrane (reviewed in Lisman et al., 2012).

Unlike the β-isoform, CaMKII-δ was strongly expressed inside bipolar cell terminals of the OFF and ON layer but was also found outside these structures (Figures 3J–R, arrows). Thus, CaMKII-β and -δ are differentially expressed in the IPL.

### CaMKII-δ but Not -β Was Found in Rod Bipolar Cells

**Figure 4:**
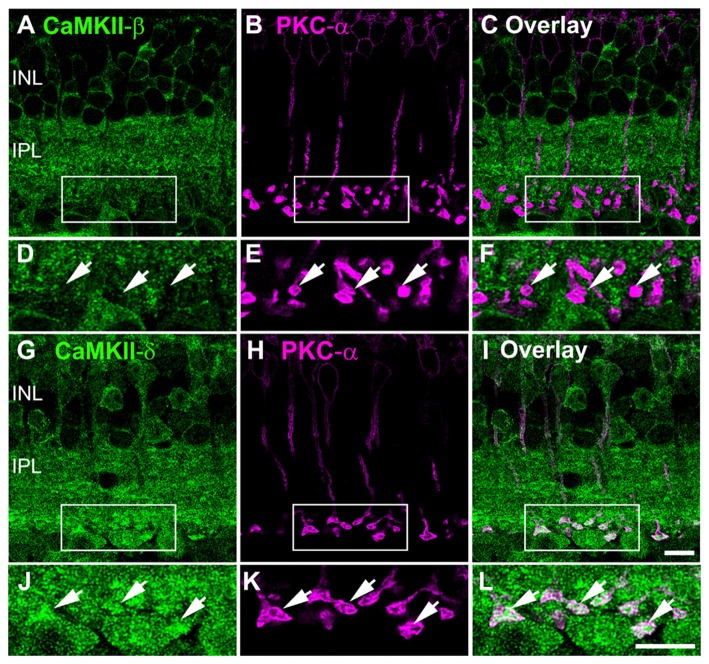
CaMKII-δ expression in rod bipolar cells. **(A–C)** Double staining of CaMKII-β and PKC-α confirmed the absence of the β-subunit in rod bipolar cells (arrows). **(D–F)** Magnified images suggest CaMKII-β expression in neurons postsynaptic to rod bipolar cells. **(G–I)** CaMKII-δ is expressed in rod bipolar cells (arrows) which is confirmed by higher magnifications **(J–L)**. Scale: 10 μm.

To further characterize the differences in distribution of CaMKII-β and -δ, we compared the expression pattern of both isoforms in rod bipolar cells. As suggested by the VGluT1 staining, PKC-α labeling confirmed that CaMKII-β was not expressed in rod bipolar cells but surrounded the terminals (Figures 4A–F). In contrast, CaMKII-δ showed strong immunoreactivity in the somata and was concentrated inside the terminals (Figures 4G–L). This confirms that rod bipolar cell terminals express CaMKII-δ but lack CaMKII-β.

### Differential Expression of CaMKII-β and -δ in Different Bipolar Cell Types

**Figure 5:**
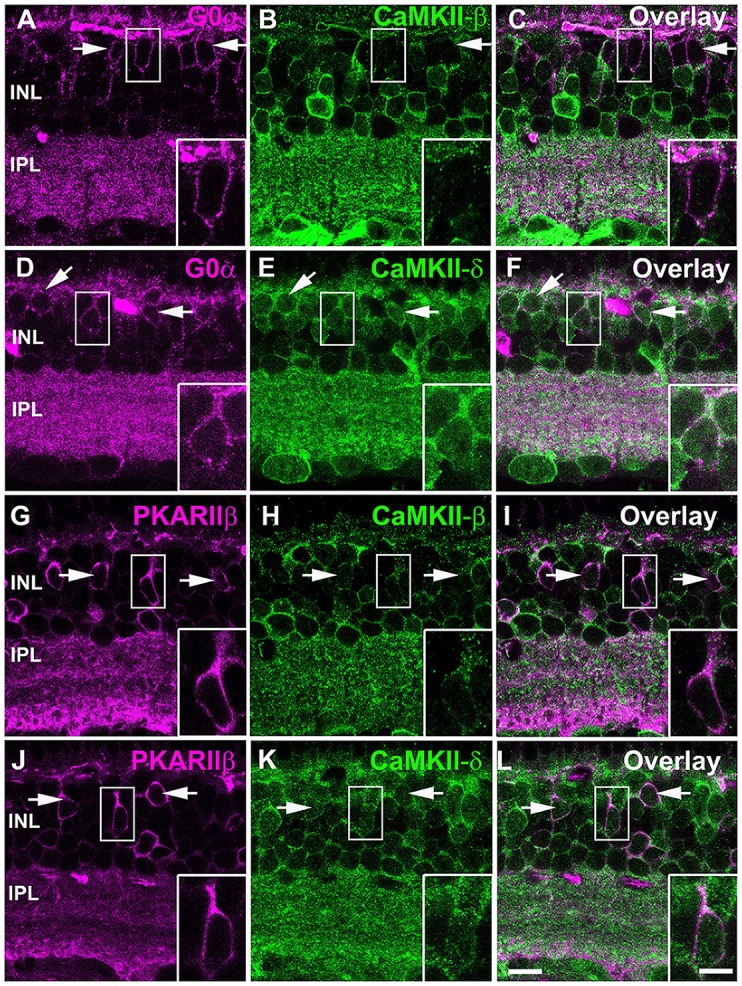
CaMKII-β and -δ expression in different types of cone biplar cells. **(A–C)** Most ON cone bipolar cells labeled with G0α (arrows) lacked CaMKII-β but expressed CaMKII-δ **(D–F)**. Type 3b OFF cone bipolar cells labeled with PKARIIβ (arrows) expressed both CaMKII-β **(G–I)** and -δ **(J–L)**. Scale: 10 μm; insets: 5 μm.

We further analyzed the expression pattern of CaMKII-β- and -δ in different cone bipolar cell types and double-labeled retinas for both CaMKII isoforms and G0α, a marker for all ON bipolar cells, and PKARIIβ, a marker for type 3b OFF bipolar cells. These stainings revealed that somata of ON bipolar cells express CaMKII-δ (Figures 5D–F) but lack CaMKII-β (Figures 5A–C). In contrast, we found coexpression of both isoforms in type 3b OFF bipolar cells (Figures 5G–L).

### CaMKII-β and -δ Colocalized with Cx36 in the IPL

**Figure 6:**
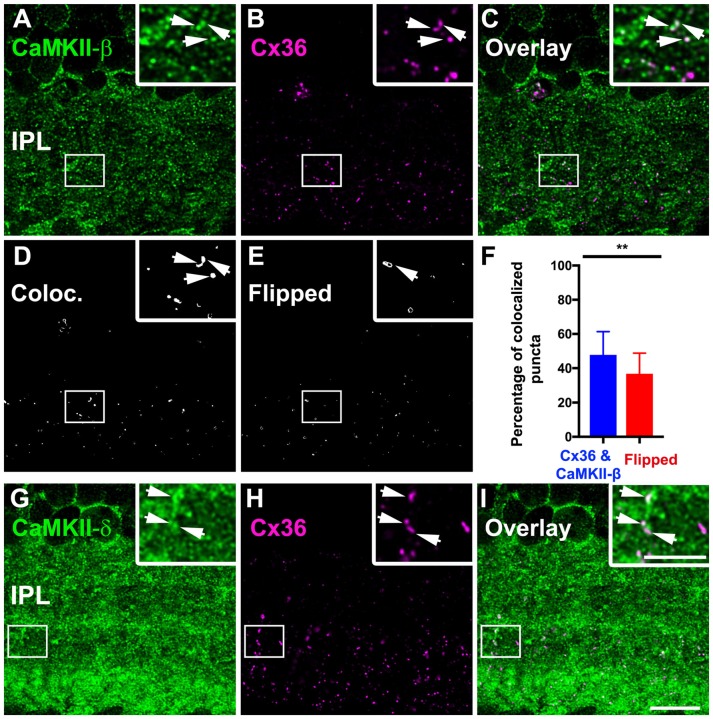
CaMKII-β and -δ colocalized with Cx36 in the IPL. **(A–C)** CaMKII-β puncta colocalized with Cx36 at individual gap junction plaques (arrows). **(D–F)** Colocalization analysis revealed significant colocalization compared to flipped control conditions (**p < 0.01, 7–10 regions of interest from three different animals, data is shown as mean ± standard deviation of the mean). **(G–I)** CaMKII-δ showed abundant overlap with Cx36 (arrows). Scale: 10 μm; insets: 5 μm.

Our next aim was to determine which CaMKII isoform is located at Cx36-containing gap junctions in the inner retina. We double-labeled cryosections with Cx36 and each CaMKII subunit and evaluated the degree of colocalization. Confocal images revealed several colocalized puncta for Cx36 and CaMKII-β (Figures 6A–C). Our statistical analysis confirmed a significant colocalization with approximately 50% colocalized puncta (46 ± 13% of total Cx36 puncta). Horizontally flipped images that were used as control showed only 36 ± 12% colocalization (Figures 6D–F), which was significantly less (*p* < 0.01, *t*-test) and confirmed true colocalization. We further examined the colocalization of Cx36 and CaMKII-δ and detected several puncta that overlapped with Cx36 (Figures 6G–I). Unfortunately, CaMKII-δ labeling in the IPL covered a very large area, preventing us from statistical verification because flipped images displayed a strong overlap as well. Finally, we analyzed the overlap for Cx36 and CaMKII-α/γ (Supplementary Figure S1) and did not observe significant colocalization, suggesting that CaMKII-β and -δ are the only subunits to associate with retinal Cx36.

### CaMKII-δ Predominantly Colocalized with Cx36 in AII Amacrine Cells

**Figure 7:**
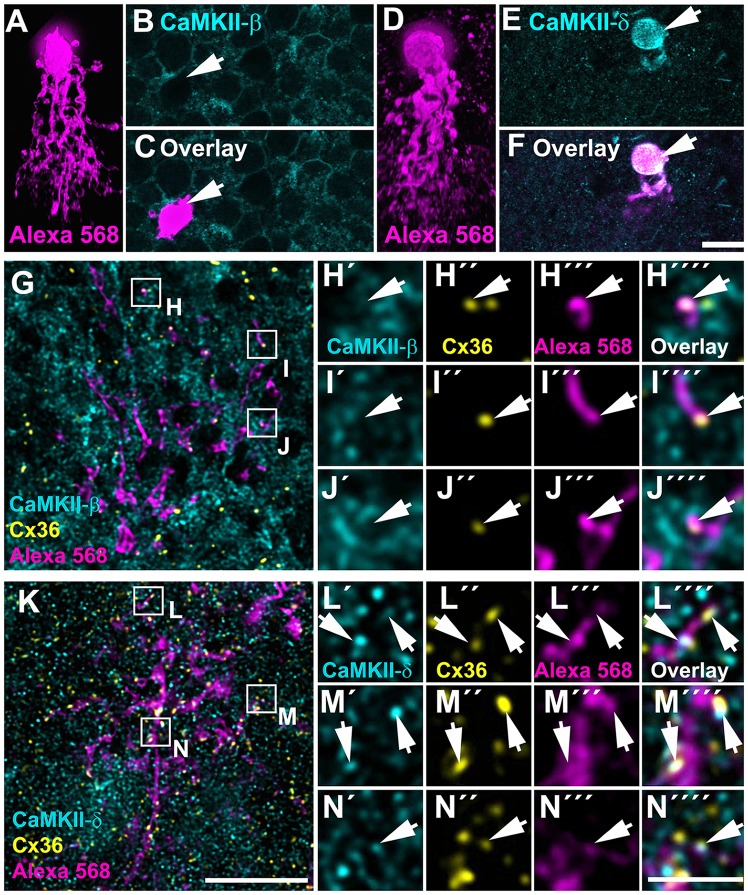
CaMKII-δ colocalized with Cx36 in AII amacrine cells. **(A–F)** Dye-injected AII amacrine cells (**A,D**, xz rotation) were labeled for CaMKII-β **(B)** and -δ **(E)**, however, only CaMKII-δ was detected in the injected cell’s soma **(F)**.** (G–N)** Whole-mount view on the arboreal dendrites of the same dye-injected AII amacrine cells labeled for either CaMKII-β **(G–J)** and CaMKII-δ **(K–N)** and Cx36. Magnified images of selected areas revealed no overlap of Cx36 and CaMKII-β **(H–J””)** but revealed strong overlap of Cx36 and CaMKII-δ puncta (arrows) on the dye-filled dendrites of the AII cell **(L–N””)**. In **(A,D)**, maximum projections of confocal stacks are shown with 84 and 80 optical sections (0.3 μm), respectively. Magnified images **(H–J,L–N)** show single confocal scans. Scale: **A–G,K**, 10 μm; **H–J,L–N**, 2.5 μm.

As outlined above, CaMKII-δ immunoreactivity in the IPL was too dense for quantification and statistical verification although colocalized puncta were clearly evident. To bypass this problem, we dye-injected AII amacrine cells (Figures 7A,D) in retinal whole-mounts and compared the expression pattern of CaMKII-β and -δ in these cells. We found that AII cell somata lacked CaMKII-β but strongly expressed CaMKII-δ (Figures 7A–F). Importantly, Cx36 puncta and CaMKII-δ often colocalized on arboreal dendrites of injected AII cells (161/619 puncta = 26% colocalization, from three injected AII cells, Figures 7K–N) whereas Cx36 and CaMKII-β were only rarely colocalized (63/787 puncta = 8% colocalization, from three injected AII cells, Figures 7G–J). This finding identifies CaMKII-δ as a major regulator of gap junctions in AII amacrine cells.

### CaMKII-δ Colocalized with Cx36 in TH2 Cells

**Figure 8:**
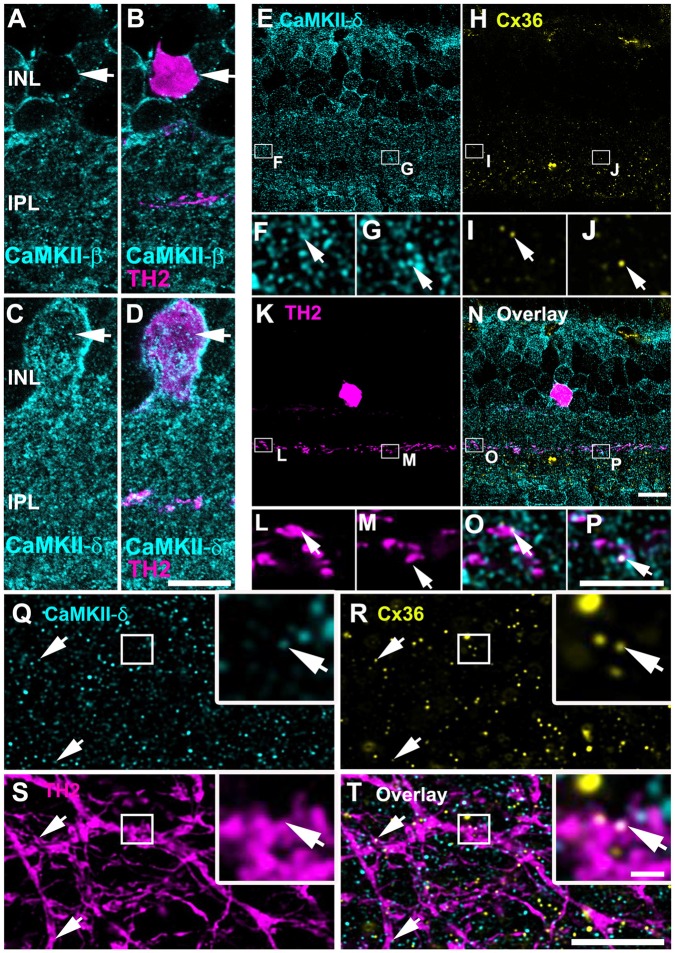
CaMKII-δ and Cx36 colocalized in tyrosine hydroxylase type2 (TH2) cells. **(A–D)** TH2 cell somata lack CaMKII-β **(A,B)** but express CaMKII-δ (arrows, **C,D**). Triple stainings in vertical slices reveal colocalization **(N–P)** of CaMKII-δ **(E–G)** and Cx36 **(H–J)** inside dendrites of TH2 cells (arrows, **K–M**). Similarly, colocalization was also detected in retinal whole-mounts **(Q–T)**. Areas marked by squares are shown in higher magnification in the insets. Scale: **A–E,H,K,N,Q–T**: 10 μm; **F,G,I,J,L,M,O,P**: 5 μm; insets in **Q–T**: 1 μm.

Our next aim was to elucidate whether CaMKII-δ is expressed in gap junction-coupled neurons other than AII amacrine cells. Previous observations described electrical coupling and Cx36-containing gap junctions in TH2 amacrine cells (Brüggen et al., 2015). In TH::GFP mice, TH2 cells can be visualized easily because they express GFP under the tyrosine hydroxylase promoter (Brüggen et al., 2015). We used these mice to examine the localization of CaMKII-β and -δ at gap junctions in TH2 cells. The expression patterns of both subunits were comparable to the ones observed in AII cells: TH2 cell somata lacked CaMKII-β but displayed strong CaMKII-δ labeling (Figures 8A–D). Moreover, stainings in vertical sections revealed that Cx36 and CaMKII-δ colocalized on the dendrites of TH2 cells, which ramify in the third IPL layer (Figures 8E–P). The degree of colocalization (85/414 = 21% of colocalization) was similar to the colocalization found for Cx36 and CaMKII-δ on AII cell processes (26%). This was quantified from retinal whole-mounts (Figures 8Q–T). Our finding suggests that TH2 cell coupling may underlie activity-dependent potentiation by CaMKII as well.

### CaMKII-β Colocalized with Cx36 at OFF Cone Bipolar Cells Dendrites

Our data so far show that CaMKII-β and -δ colocalize with Cx36 in inner retina. However, in AII and TH2 amacrine cells, we only found association with the δ subunit, suggesting that the interaction of Cx36 with certain subunits varies with different cell types.

**Figure 9:**
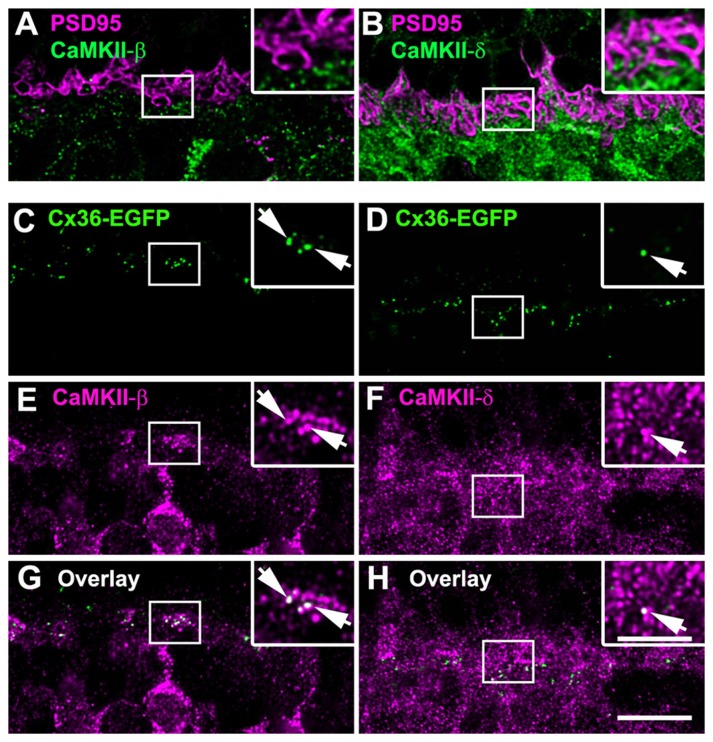
CaMKII-β colocalized with Cx36 at OFF cone bipolar cell dendrites. **(A)** CaMKII-β immunoreactivity is located underneath photoreceptor terminals. **(B)** CaMKII-δ puncta were found inside and beneath photoreceptor terminals. **(C,E,G)** CaMKII-β labeled the margin of putative OFF cone bipolar cells and colocalized with Cx36 at their dendrites (arrows). **(D,F,H)** CaMKII-δ staining showed only a few colocalized puncta with Cx36 (arrows). Images **(C–H)** show maximum projections of confocal stacks (8 optical sections, 0.2 μm thick); insets show single scans. Scale: 10 μm, insets: 5 μm.

To find out whether this is also the case in the outer retina, we further analyzed the localization of the β- and δ-isoforms and Cx36 in the OPL where most Cx36-immunoreactive puncta originate from OFF cone bipolar cells (Feigenspan et al., 2004). We first compared the overall distribution of CaMKII-β and -δ. Counterstaining with PSD-95, which labels photoreceptor terminals (Koulen et al., 1998), revealed that both isoforms are localized underneath photoreceptor terminals (Figures 9A,B). Additionally, CaMKII-δ puncta were detected inside photoreceptor terminals (Figure 9B). This localization most likely reflects CaMKII-δ expression at ON bipolar cell dendrites invaginating the photoreceptor terminal because CaMKII-δ and immunolabeling for Cacna1s (belonging to the signaling cascade in ON bipolar cells, which is located at the dendritic tips, Hasan et al., 2016) strongly overlapped (Supplementary Figure S2).

Next, we aimed to determine whether Cx36 colocalizes with CaMKII-β and CaMKII-δ in the OPL. As immunoreactivity for Cx36 was often weak in the outer retina, we used Cx36-EGFP mice to analyze this. In these mice, which express a fusion protein of Cx36 and EGFP, Cx36 puncta can be detected without antibodies. We found that Cx36-EGFP colocalized with both isoforms (Figures 9C–H). Nevertheless, overlap with CaMKII-β was much more prominent than overlap with CaMKII-δ (compare Figures 9G,H), indicating that CaMKII-β is the predominant CaMKII subunit at Cx36-containing electrical synapses in the outer retina.

## Discussion

We investigated the subunit composition of CaMKII at retinal gap junctions and revealed that β- and δ- isoforms colocalize with Cx36. Interestingly, our data point to a variable subunit composition, as CaMKII-β predominated at electrical synapses in the outer retina whereas CaMKII-δ was the predominant subunit to associate with Cx36 in AII and TH2 amacrine cells in the inner retina. Thus, although gap junctions of several retinal neurons require the same enzyme, they may recruit different variants of it in a cell type-specific manner.

As shown before in many different brain regions (Lisman et al., 2002), CaMKII-β is located in the retina at the postsynaptic side of glutamatergic synapses; in contrast, we detected CaMKII-δ inside the glutamatergic bipolar cell terminals, suggesting that the different isoforms fulfill different functions in the retina.

### CaMKII-δ Regulates Cx36-containing Gap Junctions on AII and TH2 Amacrine Cells

Several previous reports demonstrated the interaction of CaMKII and Cx36 in neurons (Alev et al., 2008; Kothmann et al., 2012; Turecek et al., 2014). In the inferior olive, electrical coupling is strengthened by activation of NMDA receptors and CaMKII-mediated phosphorylation of Cx36 (Turecek et al., 2014). In the retina, a similar pathway is active in AII amacrine cells, the key interneurons of the most sensitive rod pathway. Kothmann et al. (2012) localized activated CaMKII at Cx36-containing gap junctions in AII amacrine cells and demonstrated that Ca^2+^ influx via NMDA receptors leads to CaMKII-mediated phosphorylation of Cx36 and to an increase in gap junctional coupling between AII cells. However, for both brain regions, it remained unclear which CaMKII isoform mediates the synaptic potentiation. Our data indicate now that in AII cells, activity-dependent phosphorylation of Cx36 is predominantly mediated by CaMKII-δ. Immunolabeling in sections and intracellular dye injections into individual AII cells showed considerable overlap between CaMKII-δ and Cx36. Although CaMKII-α and -β are well characterized for their function in synaptic plasticity and interaction with NMDA receptors (Sanhueza et al., 2011), only CaMKII-β was weakly expressed at AII gap junctions (Figure 7, Supplementary Figure S3). As NMDA receptor/Cx36 complexes may exist in many other brain regions (Kothmann et al., 2012), it will be interesting to see whether it is always CaMKII-δ and maybe -β that link glutamate-mediated excitation to potentiation of electrical coupling.

Interestingly, CaMKII-δ was not found at all Cx36-containing gap junctions in the AII cell. Many Cx36 puncta were unlabeled. This may suggest that CaMKII-δ regulates only a subset of AII gap junctions, most likely the AII-AII gap junctions, which were shown to depend on NMDA receptor activity (Kothmann et al., 2012). This adds to the previous notion that the two sets of AII gap junctions (homocellullar AII-AII and heterocellular AII-ON cone bipolar cell gap junctions) are fundamentally different (Mills and Massey, 1995; Anderson et al., 2011; Meyer et al., 2014, 2016). Evidence was provided for example that AII-ON bipolar cell gap junctions are modulated by NO and cGMP whereas AII-AII gap junctions are not (Mills and Massey, 1995; Xin and Bloomfield, 1999).

We detected CaMKII-δ expression not only in AII cells but also at Cx36-containing gap junctions in TH2 cells of the TH::GFP retina. TH2 cells are GABAergic wide-field amacrine cells which depolarize in response to light ON and OFF (Knop et al., 2011) and are weakly coupled by Cx36 (Brüggen et al., 2015). It is tempting to speculate that these cells may also regulate their gap junctional network in an activity-dependent manner. Indeed, Kalloniatis et al. (2016) showed NMDA activation in calretinin-positive amacrine cells stratifying in the middle of the IPL that may well represent TH2 cells (see Figure 1E in Knop et al., 2011).

### CaMKII-β Regulates Cx36-containing Gap Junctions on OFF Bipolar Cells

In the outer retina, CaMKII-β seems to be the predominant isoform at Cx36-containing gap junctions. Its overlap with Cx36 was stronger than that of the δ-subunit. A previous study localized GluA1 clusters in close proximity to Cx36 at flat contacts of mouse OFF bipolar cells (Feigenspan et al., 2004). Also, the juxtaposition of GluA4 and Cx36 in the human outer retina was reported (Kántor et al., 2016). This arrangement resembles the organization of mixed synapses, at which glutamatergic activity modulates the strength of the electrical synapse (Pereda, 2014). GluA1 receptors, which are also a target of CaMKII (Barria et al., 1997; Mammen et al., 1997), may provide the Ca^2+^ source that leads to CaMKII activation and subsequent phosphorylation of Cx36. This pathway would tie the extent of OFF bipolar cell coupling to photoreceptor depolarization. Although the function of gap junctional coupling of OFF bipolar cells is not entirely clear yet, it was suggested that coupling decreases the dispersion of signals (Umino et al., 1994) and increases the signal-to-noise ratio (Völgyi et al., 2013), similar to AII-AII coupling (Dunn et al., 2006). Our data now suggest that the extent of OFF bipolar cell coupling may show a similar light dependence.

### Functional Implications for Gap Junction Regulation

The presence of distinct CaMKII subunits at retinal gap junctions raises the question whether this localization has a physiological significance for the regulation of Cx36. The four CaMKII isoforms differ in actin (Hoffman et al., 2013) and Ca^2+^/calmodulin binding affinity (Gaertner et al., 2004), and Ca^2+^/calmodulin dependence for autophosphorylation (Gaertner et al., 2004). For instance, CaMKII-β shows a higher Ca^2+^/calmodulin binding affinity than CaMKII-δ and may thus respond to smaller changes in intracellular Ca^2+^ levels. In this way, the differential expression of the CaMKII-β and -δ isoforms may reflect the unique properties and demands of the gap junctional network they regulate.

However, one may also argue that the differential expression of CaMKII isoforms may not represent an adaptation for the regulation of electrical synapses but may be necessary for other cellular processes, like overall Ca^2+^ homoeostasis in the cell. In this case, the subunit composition of CaMKII at the gap junction may simply depend on the cell type and its role in the retinal circuitry. Yet, the finding that individual OFF bipolar cell types express both CaMKII isoforms and target them into different compartments argues against this hypothesis. Therefore, it seems reasonable to assume that CaMKII expression differs between cell types to regulate gap junctional networks differentially even if they employ the same connexin.

### Differential Expression of CaMKII-δ and -β at Glutamatergic Synapses

CaMKII-δ and -β also showed a differential expression at glutamatergic synapses in the retina. CaMKII-δ was found presynaptically, in photoreceptor and bipolar cell terminals, whereas CaMKII-β was detected postsynaptically, in bipolar cell dendrites (see above) and putative amacrine and ganglion cell processes. The abundance of CaMKII-δ in terminals containing ribbon synapses suggests a role in neurotransmitter release. Indeed, CaMKII was found to be associated with synaptic ribbons (Uthaiah and Hudspeth, 2010) and to phosphorylate syntaxin 3B, a protein important for synaptic vesicle exocytosis at ribbon synapses (Liu et al., 2014).

In contrast, we identified CaMKII-β mainly at the postsynaptic side of glutamatergic synapses in the retina. This localization most likely reflects its function in enhancing transmission by phosphorylation or recruitment of AMPA receptors (reviewed in Lisman et al., 2002).

### Potential Function of CaMKII-α in Starburst Amacrine Cells

Strong expression of CaMKII-α was found in starburst amacrine cells. This is in line with a previous report from rat retina (Ochiishi et al., 1994). We can only speculate on CaMKII-α activation and function in starburst cells, which are involved in computing direction selectivity and release both GABA and acetylcholine as neurotransmitters. Calcium, necessary to activate CaMKII, may enter the starburst cells through AMPA receptors (Firth et al., 2003) and in turn CaMKII-α may play a role in neurotransmitter release. However, given the strong expression in starburst somata and dendrites, it seems likely that CaMKII-α fulfills many different roles in these cells. Yet, as starburst cells are not coupled by gap junctions, modulating electrical synapses is not one of them.

In summary, our results show that the distribution of CaMKII isoforms in the retina is more complex than previously realized, indicating that Ca^2+^-dependent signaling pathways are tightly controlled in the vertebrate retina.

## Author Contributions

KD, ST, UJ-B designed experiments; ST and SCY performed experiments and prepared all figures. ST wrote a first draft of the manuscript which was revised by KD. All contributed to the interpretation of data; all authors edited and commented on the manuscript and KD finalized it.

## Conflict of Interest Statement

The authors declare that the research was conducted in the absence of any commercial or financial relationships that could be construed as a potential conflict of interest.

## Footnotes

^1^http://fiji.sc/

## References

[B1] Alev C. Urschel S. Sonntag S. Zoidl G. Fort A. G. Höher T.. (2008). The neuronal connexin36 interacts with and is phosphorylated by CaMKII in a way similar to CaMKII interaction with glutamate receptors. Proc. Natl. Acad. Sci. U S A 105, 20964–20969. 10.1073/pnas.0805408105 19095792 PMC2605416

[B2] Anderson J. R. Jones B. W. Watt C. B. Shaw M. V. Yang J.-H. Demill D.. (2011). Exploring the retinal connectome. Mol. Vis. 17, 355–379. 21311605 PMC3036568

[B3] Barria A. Derkach V. Soderling T. (1997). Identification of the Ca^2+^/calmodulin-dependent protein kinase II regulatory phosphorylation site in the α-amino-3-hydroxyl-5-methyl-4-isoxazole-propionate-type glutamate receptor. J. Biol. Chem. 272, 32727–32730. 10.1074/jbc.272.52.32727 9407043

[B5] Bloomfield S. A. Völgyi B. (2004). Function and plasticity of homologous coupling between AII amacrine cells. Vis. Res. 44, 3297–3306. 10.1016/j.visres.2004.07.012 15535997

[B4] Bloomfield S. A. Völgyi B. (2009). The diverse functional roles and regulation of neuronal gap junctions in the retina. Nat. Rev. Neurosci. 10, 495–506. 10.1038/nrn2636 19491906 PMC3381350

[B6] Brüggen B. Meyer A. Boven F. Weiler R. Dedek K. (2015). Type 2 wide-field amacrine cells in TH::GFP mice show a homogenous synapse distribution and contact small ganglion cells. Eur. J. Neurosci. 41, 734–747. 10.1111/ejn.12813 25546402

[B7] Christie J. M. Bark C. Hormuzdi S. G. Helbig I. Monyer H. Westbrook G. L. (2005). Connexin36 mediates spike synchrony in olfactory bulb glomeruli. Neuron 46, 761–772. 10.1016/j.neuron.2005.04.030 15924862

[B8] Deans M. R. Volgyi B. Goodenough D. A. Bloomfield S. A. Paul D. L. (2002). Connexin36 is essential for transmission of rod-mediated visual signals in the mammalian retina. Neuron 36, 703–712. 10.1016/s0896-6273(02)01046-2 12441058 PMC2834592

[B9] Dedek K. Schultz K. Pieper M. Dirks P. Maxeiner S. Willecke K.. (2006). Localization of heterotypic gap junctions composed of connexin45 and connexin36 in the rod pathway of the mouse retina. Eur. J. Neurosci. 24, 1675–1686. 10.1111/j.1460-9568.2006.05052.x 17004931

[B10] Del Corsso C. Iglesias R. Zoidl G. Dermietzel R. Spray D. C. (2012). Calmodulin dependent protein kinase increases conductance at gap junctions formed by the neuronal gap junction protein connexin36. Brain Res. 1487, 69–77. 10.1016/j.brainres.2012.06.058 22796294 PMC4355912

[B11] Dunn F. A. Doan T. Sampath A. P. Rieke F. (2006). Controlling the gain of rod-mediated signals in the mammalian retina. J. Neurosci. 26, 3959–3970. 10.1523/JNEUROSCI.5148-05.2006 16611812 PMC6673884

[B12] Feigenspan A. Janssen-Bienhold U. Hormuzdi S. Monyer H. Degen J. Söhl G.. (2004). Expression of connexin36 in cone pedicles and OFF-cone bipolar cells of the mouse retina. J. Neurosci. 24, 3325–3334. 10.1523/JNEUROSCI.5598-03.2004 15056712 PMC6730041

[B13] Firth S. I. Li W. Massey S. C. Marshak D. W. (2003). AMPA receptors mediate acetylcholine release from starburst amacrine cells in the rabbit retina. J. Comp. Neurol. 466, 80–90. 10.1002/cne.10880 14515241 PMC3341736

[B14] Flores C. E. Cachope R. Nannapaneni S. Ene S. Nairn A. C. Pereda A. E. (2010). Variability of distribution of Ca^2+^/calmodulin-dependent kinase II at mixed synapses on the mauthner cell: colocalization and association with connexin 35. J. Neurosci. 30, 9488–9499. 10.1523/JNEUROSCI.4466-09.2010 20631177 PMC2945303

[B15] Gaertner T. R. Kolodziej S. J. Wang D. Kobayashi R. Koomen J. M. Stoops J. K.. (2004). Comparative analyses of the three-dimensional structures and enzymatic properties of α, β, γ, and δ isoforms of Ca^2+^-calmodulin-dependent protein kinase II. J. Biol. Chem. 279, 12484–12494. 10.1074/jbc.M313597200 14722083

[B16] Güldenagel M. Ammermüller J. Feigenspan A. Teubner B. Degen J. Söhl G.. (2001). Visual transmission deficits in mice with targeted disruption of the gap junction gene connexin36. J. Neurosci. 21, 6036–6044. 11487627 10.1523/JNEUROSCI.21-16-06036.2001 PMC6763178

[B17] Han Y. Massey S. C. (2005). Electrical synapses in retinal ON cone bipolar cells: subtype-specific expression of connexins. Proc. Natl. Acad. Sci. U S A 102, 13313–13318. 10.1073/pnas.0505067102 16150718 PMC1201596

[B18] Hasan N. Ray T. A. Gregg R. G. (2016). CACNA1S expression in mouse retina: novel isoforms and antibody cross-reactivity with GPR179. Vis. Neurosci. 33:E009. 10.1017/S0952523816000055 27471951 PMC6815669

[B19] Hilgen G. von Maltzahn J. Willecke K. Weiler R. Dedek K. (2011). Subcellular distribution of connexin45 in OFF bipolar cells of the mouse retina. J. Comp. Neurol. 519, 433–450. 10.1002/cne.22526 21192077

[B20] Hoffman L. Farley M. M. Waxham M. N. (2013). Calcium-calmodulin-dependent protein kinase II isoforms differentially impact the dynamics and structure of the actin cytoskeleton. Biochemistry 52, 1198–1207. 10.1021/bi3016586 23343535 PMC3578116

[B21] Hormuzdi S. G. Pais I. LeBeau F. E. Towers S. K. Rozov A. Buhl E. H.. (2001). Impaired electrical signaling disrupts γ frequency oscillations in connexin 36-deficient mice. Neuron 31, 487–495. 10.1016/s0896-6273(01)00387-7 11516404

[B22] Kalloniatis M. Nivison-Smith L. Chua J. Acosta M. L. Fletcher E. L. (2016). Using the rd1 mouse to understand functional and anatomical retinal remodelling and treatment implications in retinitis pigmentosa: a review. Exp. Eye Res. 150, 106–121. 10.1016/j.exer.2015.10.019 26521764

[B23] Kántor O. Benkő Z. Énzsöly A. Dávid C. Naumann A. Nitschke R.. (2016). Characterization of connexin36 gap junctions in the human outer retina. Brain Struct. Funct. 221, 2963–2984. 10.1007/s00429-015-1082-z 26173976

[B24] Kim K. Lakhanpal G. Lu H. E. Khan M. Suzuki A. Hayashi M. K.. (2015). A temporary gating of actin remodeling during synaptic plasticity consists of the interplay between the kinase and structural functions of CaMKII. Neuron 87, 813–826. 10.1016/j.neuron.2015.07.023 26291163 PMC4548268

[B25] Knop G. C. Feigenspan A. Weiler R. Dedek K. (2011). Inputs underlying the ON-OFF light responses of type 2 wide-field amacrine cells in TH::GFP mice. J. Neurosci. 31, 4780–4791. 10.1523/JNEUROSCI.6235-10.2011 21451016 PMC6622987

[B26] Kothmann W. W. Massey S. C. O’Brien J. (2009). Dopamine-stimulated dephosphorylation of connexin 36 mediates AII amacrine cell uncoupling. J. Neurosci. 29, 14903–14911. 10.1523/JNEUROSCI.3436-09.2009 19940186 PMC2839935

[B27] Kothmann W. W. Trexler E. B. Whitaker C. M. Li W. Massey S. C. O’Brien J. (2012). Nonsynaptic NMDA receptors mediate activity-dependent plasticity of gap junctional coupling in the AII amacrine cell network. J. Neurosci. 32, 6747–6759. 10.1523/JNEUROSCI.5087-11.2012 22593045 PMC3367513

[B28] Koulen P. Fletcher E. L. Craven S. E. Bredt D. S. Wässle H. (1998). Immunocytochemical localization of the postsynaptic density protein PSD-95 in the mammalian retina. J. Neurosci. 18, 10136–10149. 9822767 10.1523/JNEUROSCI.18-23-10136.1998 PMC6793313

[B29] Lisman J. Schulman H. Cline H. (2002). The molecular basis of CaMKII function in synaptic and behavioural memory. Nat. Rev. Neurosci. 3, 175–190. 10.1038/nrn753 11994750

[B30] Lisman J. Yasuda R. Raghavachari S. (2012). Mechanisms of CaMKII action in long-term potentiation. Nat. Rev. Neurosci. 13, 169–182. 10.1038/nrn3192 22334212 PMC4050655

[B32] Liu X. Heidelberger R. Janz R. (2014). Phosphorylation of syntaxin 3B by CaMKII regulates the formation of t-SNARE complexes. Mol. Cell. Neurosci. 60, 53–62. 10.1016/j.mcn.2014.03.002 24680688 PMC4066811

[B31] Liu L. O. Li G. McCall M. A. Cooper N. G. (2000). Photoreceptor regulated expression of Ca^2+^/calmodulin-dependent protein kinase II in the mouse retina. Mol. Brain Res. 82, 150–166. 10.1016/s0169-328x(00)00203-5 11042368

[B33] Mammen A. L. Kameyama K. Roche K. W. Huganir R. L. (1997). Phosphorylation of the α-amino-3-hydroxy-5-methylisoxazole4-propionic acid receptor GluR1 subunit by calcium/calmodulin-dependent kinase II. J. Biol. Chem. 272, 32528–32533. 10.1074/jbc.272.51.32528 9405465

[B34] Matsushita N. Okada H. Yasoshima Y. Takahashi K. Kiuchi K. Kobayashi K. (2002). Dynamics of tyrosine hydroxylase promoter activity during midbrain dopaminergic neuron development. J. Neurochem. 82, 295–304. 10.1046/j.1471-4159.2002.00972.x 12124430

[B35] Maxeiner S. Dedek K. Janssen-Bienhold U. Ammermüller J. Brune H. Kirsch T.. (2005). Deletion of connexin45 in mouse retinal neurons disrupts the rod/cone signaling pathway between AII amacrine and ON cone bipolar cells and leads to impaired visual transmission. J. Neurosci. 25, 566–576. 10.1523/JNEUROSCI.3232-04.2005 15659592 PMC6725315

[B36] Meyer A. Hilgen G. Dorgau B. Sammler E. M. Weiler R. Monyer H.. (2014). AII amacrine cells discriminate between heterocellular and homocellular locations when assembling connexin36-containing gap junctions. J. Cell Sci. 127, 1190–1202. 10.1242/jcs.133066 24463820 PMC3953814

[B37] Meyer A. Tetenborg S. Greb H. Segelken J. Dorgau B. Weiler R.. (2016). Connexin30.2: *in vitro* interaction with connexin36 in HeLa cells and expression in AII amacrine cells and intrinsically photosensitive ganglion cells in the mouse retina. Front. Mol. Neurosci. 9:36. 10.3389/fnmol.2016.00036 27303262 PMC4882342

[B38] Mills S. L. Massey S. C. (1995). Differential properties of two gap junctional pathways made by AII amacrine cells. Nature 377, 734–737. 10.1038/377734a0 7477263

[B39] Ochiishi T. Terashima T. Yamauchi T. (1994). Specific distribution of Ca^2+^/calmodulin-dependent protein kinase II α and β isoforms in some structures of the rat forebrain. Brain Res. 659, 179–193. 10.1016/0006-8993(94)90877-x 7820660

[B40] Okamoto K.-I. Narayanan R. Lee S. H. Murata K. Hayashi Y. (2007). The role of CaMKII as an F-actin-bundling protein crucial for maintenance of dendritic spine structure. Proc. Natl. Acad. Sci. U S A 104, 6418–6423. 10.1073/pnas.0701656104 17404223 PMC1851051

[B41] Pereda A. E. (2014). Electrical synapses and their functional interactions with chemical synapses. Nat. Rev. Neurosci. 15, 250–263. 10.1038/nrn3708 24619342 PMC4091911

[B42] Pereda A. E. Curti S. Hoge G. Cachope R. Flores C. E. Rash J. E. (2013). Gap junction-mediated electrical transmission: regulatory mechanisms and plasticity. Biochim. Biophys. Acta 1828, 134–146. 10.1016/j.bbamem.2012.05.026 22659675 PMC3437247

[B43] Sanhueza M. Fernandez-Villalobos G. Stein I. S. Kasumova G. Zhang P. Bayer K. U.. (2011). Role of the CaMKII/NMDA receptor complex in the maintenance of synaptic strength. J. Neurosci. 31, 9170–9178. 10.1523/JNEUROSCI.1250-11.2011 21697368 PMC3138556

[B44] Schindelin J. Arganda-Carreras I. Frise E. Kaynig V. Longair M. Pietzsch T.. (2012). Fiji: an open-source platform for biological-image analysis. Nat. Methods 9, 676–682. 10.1038/nmeth.2019 22743772 PMC3855844

[B45] Turecek J. Yuen G. S. Han V. Z. Zeng X.-H. Bayer K. U. Welsh J. P. (2014). NMDA receptor activation strengthens weak electrical coupling in mammalian brain. Neuron 81, 1375–1388. 10.1016/j.neuron.2014.01.024 24656255 PMC4266555

[B46] Umino O. Maehara M. Hidaka S. Kita S. Hashimoto Y. (1994). The network properties of bipolar-bipolar cell coupling in the retina of teleost fishes. Vis. Neurosci. 11, 533–548. 10.1017/s0952523800002443 8038127

[B47] Uthaiah R. C. Hudspeth A. J. (2010). Molecular anatomy of the hair cell’s ribbon synapse. J. Neurosci. 30, 12387–12399. 10.1523/JNEUROSCI.1014-10.2010 20844134 PMC2945476

[B48] Veruki M. L. Hartveit E. (2002). Electrical synapses mediate signal transmission in the rod pathway of the mammalian retina. J. Neurosci. 22, 10558–10566. 12486148 10.1523/JNEUROSCI.22-24-10558.2002 PMC6758447

[B49] Völgyi B. Kovács-Öller T. Atlasz T. Wilhelm M. Gábriel R. (2013). Gap junctional coupling in the vertebrate retina: variations on one theme? Prog. Retin. Eye Res. 34, 1–18. 10.1016/j.preteyeres.2012.12.002 23313713

[B50] Weinreuter M. Kreusser M. M. Beckendorf J. Schreiter F. C. Leuschner F. Lehmann L. H.. (2014). CaM Kinase II mediates maladaptive post-infarct remodeling and pro-inflammatory chemoattractant signaling but not acute myocardial ischemia/reperfusion injury. EMBO Mol. Med. 6, 1231–1245. 10.15252/emmm.201403848 25193973 PMC4287929

[B51] Xin D. Bloomfield S. A. (1999). Comparison of the responses of AII amacrine cells in the dark- and light-adapted rabbit retina. Vis. Neurosci. 16, 653–665. 10.1017/s0952523899164058 10431914

