# Supplementary material for: Differential Distribution of Retinal Ca2+/Calmodulin-Dependent Kinase II (CaMKII) Isoforms Indicates CaMKII-β and -δ as Specific Elements of Electrical Synapses Made of Connexin36 (Cx36)
Source: Front Mol Neurosci. 2017 Dec 19;10:425. doi: 10.3389/fnmol.2017.00425 (PMC5742114; doi:10.3389/fnmol.2017.00425)
Supplement: Supplementary file 1 [file Presentation_1.pdf]

Supplementary Material

Differential Distribution of Retinal Ca^{2+}/Calmodulin-Dependent Kinase II (CaMKII) Isoforms Indicates CaMKII-β and -δ as Specific Elements of Electrical Synapses Made of Connexin36 (Cx36)

Stephan Tetenborg[1], Shubhash Chandra Yadav[1], Sheriar Gustad Hormuzdi[2], Hannah Monyer[3], Ulrike Janssen-Bienhold[4, 5], Karin Dedek[1, 5*]

[1]Animal Navigation/Neurosensorics, Institute for Biology and Environmental Sciences, University of Oldenburg, Oldenburg, Germany

[2]Division of Neuroscience, Ninewells Hospital and Medical School, University of Dundee, Dundee, UK

[3]Cancer Research Center (DKFZ), Heidelberg, Germany

[4]Visual Neuroscience, Dept. of Neuroscience, University of Oldenburg, Oldenburg, Germany

[5]Research Center Neurosensory Science, University of Oldenburg, Oldenburg, Germany

***Correspondence:**
Karin Dedek
karin.dedek@uni-oldenburg.de

1 Supplementary Figures

1.1 Supplementary Figures

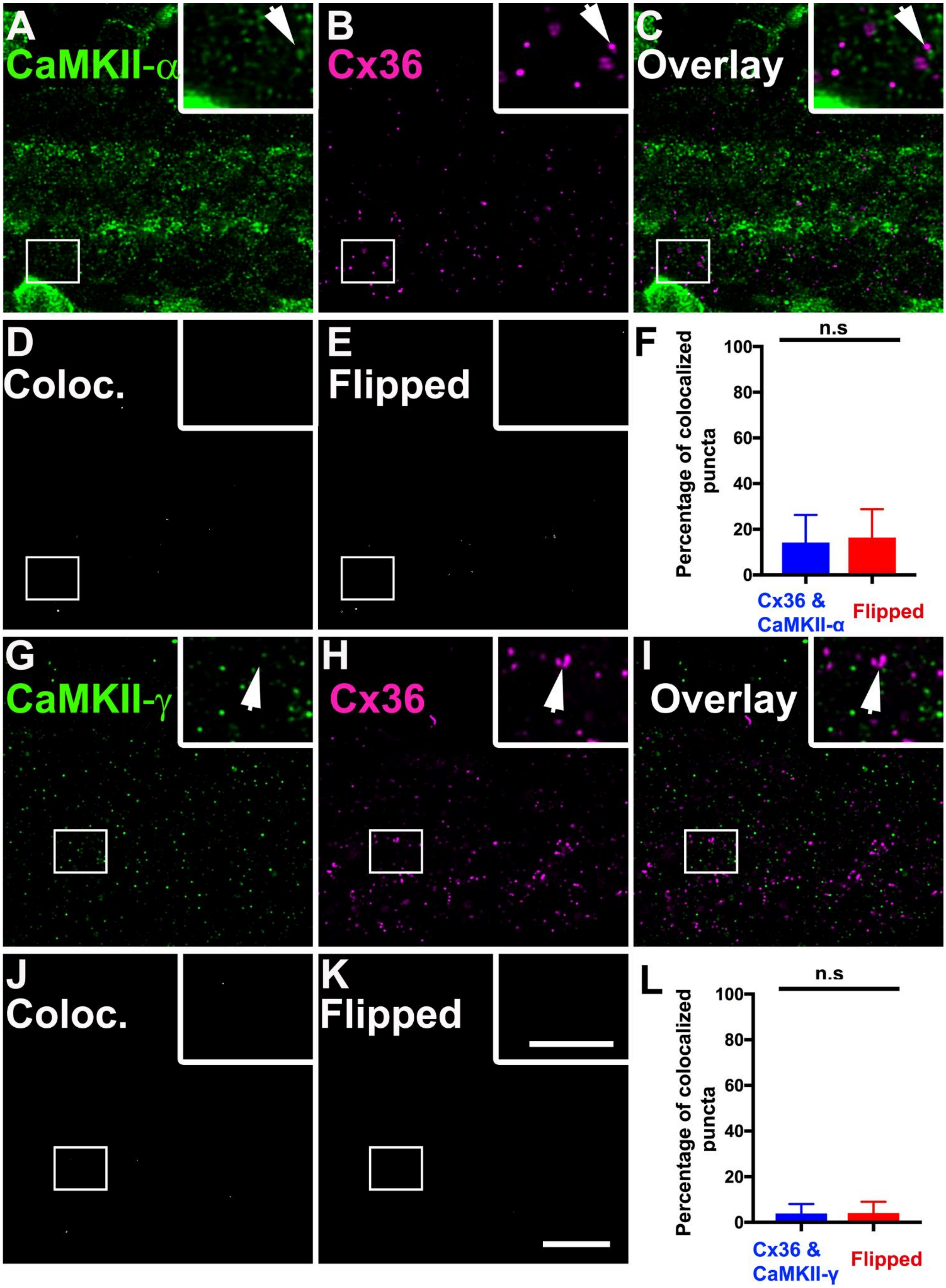
A
CaMKII-α
B
Cx36
C
Overlay
D
Coloc.
E
Flipped
F
n.s
Percentage of colocalized puncta
100
80
60
40
20
0
Cx36 & CaMKII-α
Flipped
G
CaMKII-γ
H
Cx36
I
Overlay
J
Coloc.
K
Flipped
L
n.s
Percentage of colocalized puncta
100
80
60
40
20
0
Cx36 & CaMKII-γ
Flipped

Supplementary Figure 1. CaMKII-α and -γ did not colocalize with Cx36 in the inner plexiform layer (IPL). (A-C) CaMKII-α puncta did not colocalize with Cx36 at individual gap junction plaques (arrows). (**D-F**) Colocalization analysis showed no significant differences between original and flipped control images. **(G-L)** CaMKII-γ puncta did not overlap with Cx36 either (arrows). Data in F and L are shown as mean ± standard deviation. Scale: 10 µm. Insets: 5 µm.

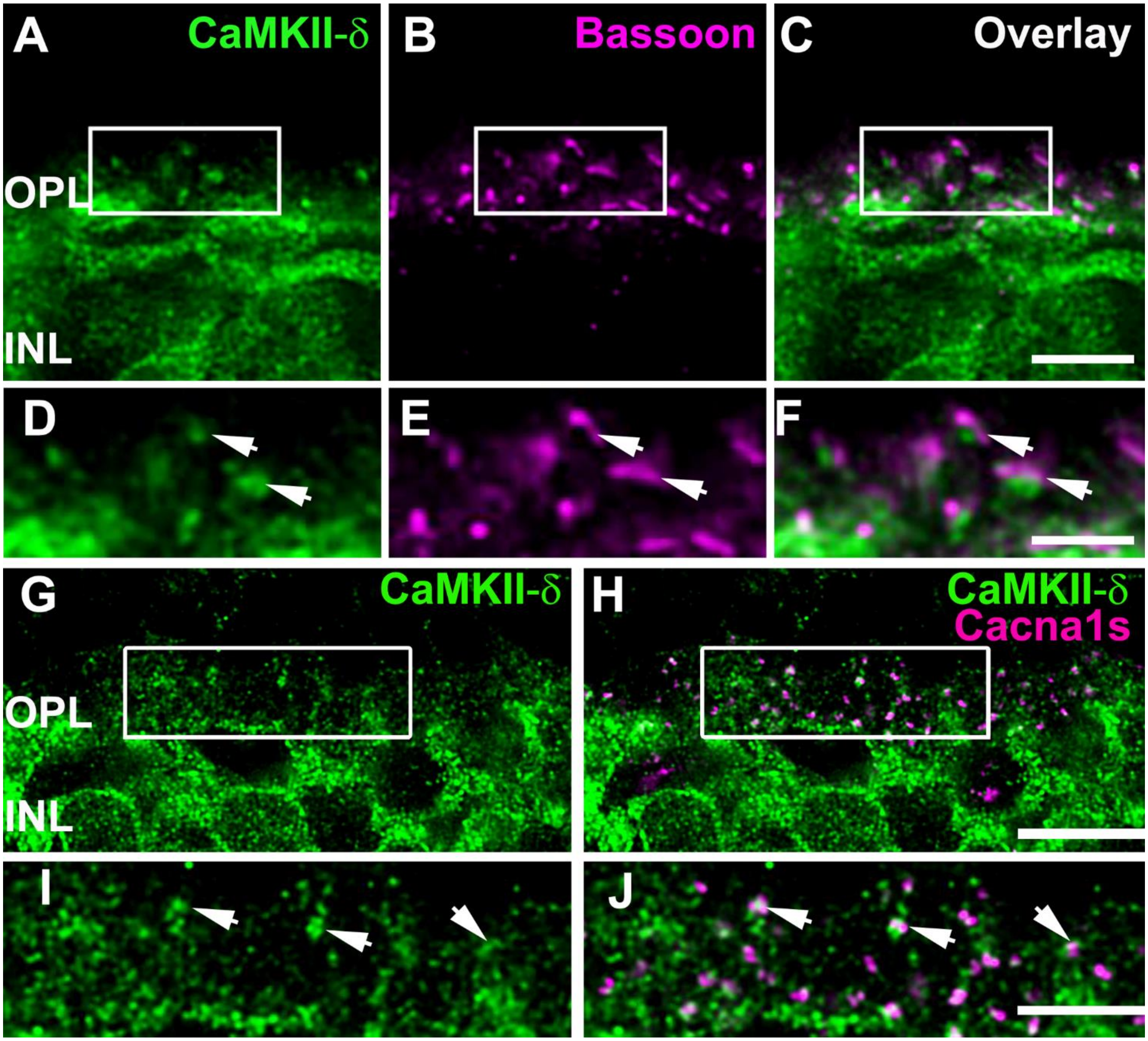

Supplementary Figure 2. CaMKII-δ was expressed at invaginating ON bipolar cell contacts. (**A-F**) CaMKII-δ and the ribbon marker bassoon were associated, but did not colocalize in the distal outer plexiform layer (arrow). (**G-J**) CaMKII-δ labeling overlapped with the mGluR6 signaling complex, labeled by antibodies directed against Cacna1s (Hasan et al., 2016). Scale: C, 10 µm; H, J, 5 µm; F, 2.5 µm.

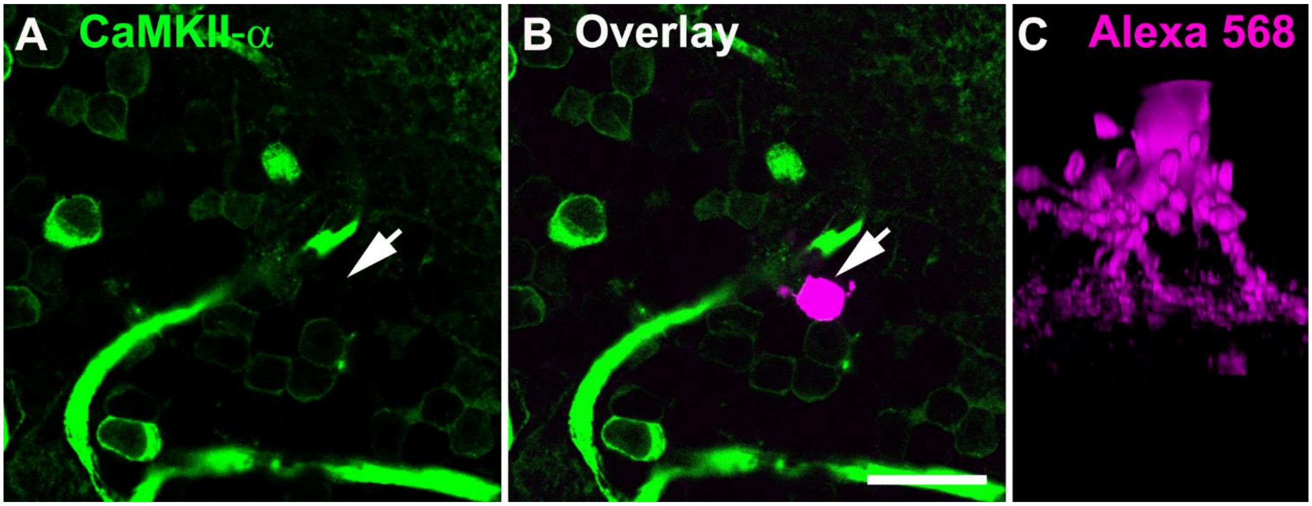

Supplementary Figure 3. CaMKII-α was absent from AII amacrine cells. (**A-C**) A retina with a dye-injected AII amacrine cell **(B, C)** was stained for CaMKII-α **(A)**. However, CaMKII-α was absent from the dye-filled cell (arrow). The image in C shows the rotated xz-projection of the injected cell in B (67 sections, 0.3 µm thick). Scale: 20 µm.

2 Supplementary References

Hasan, N., Ray, T. A., and Gregg, R. G. (2016). CACNA1S expression in mouse retina: Novel isoforms and antibody cross-reactivity with GPR179. *Vis. Neurosci.* 33, E009. doi:10.1017/S0952523816000055.
